# *Rhizobium* Promotes Non-Legumes Growth and Quality in Several Production Steps: Towards a Biofertilization of Edible Raw Vegetables Healthy for Humans

**DOI:** 10.1371/journal.pone.0038122

**Published:** 2012-05-31

**Authors:** Paula García-Fraile, Lorena Carro, Marta Robledo, Martha-Helena Ramírez-Bahena, José-David Flores-Félix, María Teresa Fernández, Pedro F. Mateos, Raúl Rivas, José Mariano Igual, Eustoquio Martínez-Molina, Álvaro Peix, Encarna Velázquez

**Affiliations:** 1 Departamento de Microbiología y Genética, Universidad de Salamanca, Salamanca, Spain; 2 Centro Hispano-Luso de Investigaciones Agrarias, Salamanca, Spain; 3 Instituto de Recursos Naturales y Agrobiología, Consejo Superior de Investigaciones Científicas, Salamanca, Spain; 4 Unidad Asociada Grupo de Interacción planta-microorganismo, Universidad de Salamanca–Consejo Superior de Investigaciones Científicas, Salamanca, Spain; University of Wisconsin-Milwaukee, United States of America

## Abstract

The biofertilization of crops with plant-growth-promoting microorganisms is currently considered as a healthy alternative to chemical fertilization. However, only microorganisms safe for humans can be used as biofertilizers, particularly in vegetables that are raw consumed, in order to avoid sanitary problems derived from the presence of pathogenic bacteria in the final products. In the present work we showed that *Rhizobium* strains colonize the roots of tomato and pepper plants promoting their growth in different production stages increasing yield and quality of seedlings and fruits. Our results confirmed those obtained in cereals and alimentary oil producing plants extending the number of non-legumes susceptible to be biofertilized with rhizobia to those whose fruits are raw consumed. This is a relevant conclusion since safety of rhizobia for human health has been demonstrated after several decades of legume inoculation ensuring that they are optimal bacteria for biofertilization.

## Introduction

In May 2011, the infection by the strain Shiga toxin-producing *Escherichia coli* O104:H4 in Germany raised the alarm regarding consumption of raw vegetables fertilised or germinated with organic products [1]. Although food-producing animals represent the most important source for the entry of this strain in the food chain [2], human infection with this bacterium can also occur through the inadvertent ingestion of fecal matter with contaminated foods [1]. This must be a wake up call about the microorganisms applied to the crops and especially to vegetables that are raw consumed. The current concern about food quality and human health has lead to a search for alternatives for substitution of agrochemicals by biological products. Among them, plant growth promoting rhizobacteria (PGPR) are an attractive tool for this purpose [3], [4]. These microorganims can influence the plant hormonal balance producing compounds such as the phytohormone indole acetic acid or the enzyme ACC deaminase involved in the metabolism of 1-aminocyclopropane-1-carboyclic acid (ACC), a precursor of ethylene. They can also mobilize nutrients to the plants such as phosphorous via solubilization of soil insoluble phosphates. Microorganisms presenting one or several of these mechanisms can directly promote plant growth. Also, some rhizospheric microorganisms produce microbial inhibitory compounds such as siderophores, Fe^+3^ ion-chelating molecules, that inhibit the growth of phytopathogens in soils with low content of this ion promoting indirectly the plant growth [3], [4].

Nevertheless, although the rhizosphere is a good source of plant growth promoting bacteria it is also a reservoir of human pathogens [5]. For example, *Burkholderia cepacia* complex, *Pseudomonas aeruginosa* and genus *Acinetobacter* contain plant growth promoting strains [6], [7], [8], [9], [10], but they should not be used as biofertilizers because these bacteria can also cause severe human infections [6], [11], [12].

Additionally, different species from family *Enterobacteriaceae* are being investigated as plant growth promoters including the human pathogen *Klebsiella pneumoniae* and the emerging plant pathogen *Pantoea ananatis* [13], [14], [15]. However, the use enterobacteria as biofertilizers involves a risk since it has been reported that clinic and plant associated strains of *Pantoea agglomerans* have indistinguishable virulence potential [16]. The risk is particularly high in the case of compost tea that is used as a spray or soil drench to promote plant growth and for control of foliar and root diseases in several plants [17] such as pepper and tomato [18], [19]. However, when compost teas are added by foliar application, pathogenic enterobacteria can persist in the food. For instance, it has been reported in the case of the strain *E. coli* O157:H7 [17] that can re-grow in compost under certain conditions [20]. This strain is an important human pathogen and has been increasingly linked to foodborne illness associated with fresh products, particularly in leafy greens such as lettuce [21].

Therefore, it is necessary to know the beneficial and harmful effects of microorganisms before their use as biofertilizers in order to use only microorganisms safe for human health, not only for the consumers or end users but also for handlers during the biofertilizer manufacturing. Strains from *Azospirillum*, *Gluconacetobacter*, *Bacillus* or *Azotobacter* are currently commercialised as biofertilizers for non-legumes without adverse effects reported for humans [22]. Nevertheless, up to date there are no commercial biofertilizers for non-legumes based on rhizobia although their safety for humans has been proven after decades of legume inoculation [3], [22] and they have potential as non-legume plant growth promoters [3], [4]. Specifically, the ability of rhizobia to promote the growth of cereals such as maize, barley and rice is well known [23], [24], [25], [26], [27] and also for other plants whose seeds are used to produce alimentary oil such as canola or sunflower [28], [29] (Table 1). Nevertheless, due to their safety for human health, rhizobia are particularly interesting in the biofertilization of raw consumed non-legumes. Currently we have data about growth promotion of vegetables edible as raw leaves such as lettuce [23], [28] and as raw roots such as radishes [30] (Table 1). However there is a lack of data on the effect of rhizobia in non-legumes rendering raw consumed fruits.

**Table 1:** Rhizobia able to promote non-legume plant growth.

Species	Biovar	Strains	Non-legume plant	Edible or feeding useful part	*in vitro* PGP mechanisms	Reference
*Rhizobium leguminosarum*	phaseoli	P31	maize	seeds	P solubilization siderophores IAA	[23]
*Rhizobium leguminosarum*	phaseoli	R1	lettuce	leaves	P solubilization siderophores IAA	[23]
*Rhizobium leguminosarum*	phaseoli	RRE6	rice	seeds	no data	[27]
*Rhizobium leguminosarum*	trifolii	E11	rice	seeds	P solubilization IAA	[26]
*Rhizobium leguminosarum*	trifolii	ANU843	rice	seeds	no data	[27]
*Rhizobium leguminosarum*	viciae	VF39SM	canola and lettuce	seeds and leaves	IAA	[28]
*Rhizobium leguminosarum*	phaseoli	TPV08	tomato and pepper	fruits	siderophores IAA	this study
*Rhizobium leguminosarum*	trifolii	PETP01	tomato and pepper	fruits	IAA	this study
*Rhizobium alamii*	unknown	YAS34	sunflowers	seeds	no data	[29]
*Rhizobium etli*	phaseoli	CFN42 CFNEM5-1	maize	seeds	no data	[24]
*Mesorhizobium mediterraneum*	ciceri	PECA21	barley	seeds	P solubilization	[25]
*Bradyrhizobium japonicum*	unknown	soy 213 TAL 629	radishes	roots	siderophores	[30]

PGP: Plant Growth Promotion. IAA: indole acetic acid.

Therefore, the aim of this work was to analyse the effect of *Rhizobium* inoculation on tomato and pepper, two economically very important vegetables whose raw fruits are consumed worldwide. Our results demonstrate that *Rhizobium* is able to colonize the roots of tomato and pepper promoting their growth in different steps of production. These results together with those previously found in other works showed that the use of rhizobia is a reliable method for non-legume biofertilization that should be further explored.

## Results

### Phylogenetic location of Rhizobium strains

**Figure 1:**
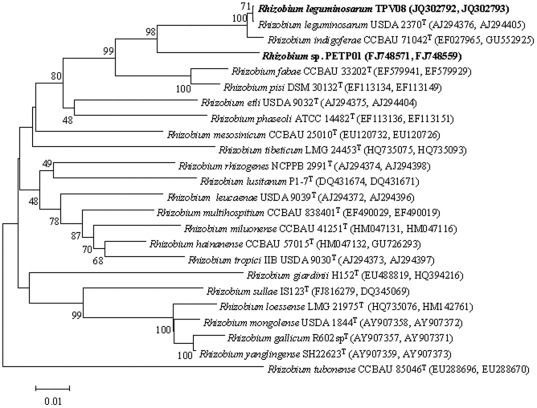
Neighbour-joining phylogenetic tree based on concatenated *recA* and *atpD* gene sequences (520 and 500 nt, respectively) showing the position of strains PETP01 and TPV08. Bootstrap values calculated for 1000 replications are indicated. Bar, 1 nt substitution per 100 nt.

The strains used in this study, PETP01 and TPV08, belong to genus *Rhizobium* and more specifically to the phylogenetic group of *R. leguminosarum* according to the results of the analysis of concatenated *recA* and *atpD* genes (figure 1). Nevertheless, they belong to different subphyla since strain TPV08 have identical *recA* and *atpD* gene sequences that the type strain of *R. leguminosarum* USDA 2307^T^ whereas in the case of strain PETP01 the identity values were lower than 94% in both genes. The results of *nodC* gene phylogenetic analysis showed that PETP01 and TPV08 also belong to different biovars, trifolii and phaseoli, respectively (figure S1).

### Rhizobium colonizes tomato and pepper root surfaces

**Figure 2:**
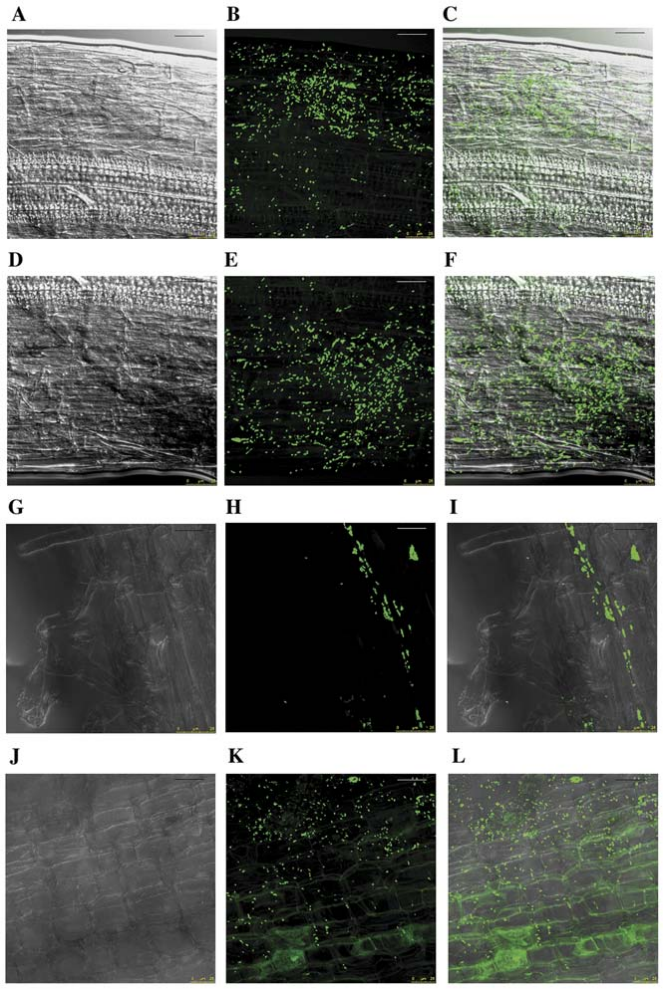
Confocal laser scanning micrographs of tomato and pepper seedling roots 9 days after inoculation with GFP-tagged cells of TPV08 and PETP01 strains. Images obtained in A, D, G and J by transmitted light in bright field mode, B, E, H and K in epifluorescence and C, F, I and L in projection. A–C: inoculation of TPV08 strain in tomato. D–F: Inoculation of PETP01 strain in tomato. G–I: inoculation of TPV08 in pepper. J–L: inoculation of PETP01 in pepper. The micrographs show the ability of strains PETP01 and TPV08 to colonize the roots surfaces. Bar, 25 µm.

GFP tagged strains PETP01 and TPV08 were inoculated on tomato and pepper seedling roots which were observed daily with a fluorescence microscope showing that the attachment gradually increased until it peaked 9 days after inoculation (data not shown). At this point the root tissues were observed with confocal microscopy that showed bacteria firmly attached to tomato and pepper root surfaces (figure 2). Typical microcolonies of biofilm initiation can be observed, especially colonizing intercellular spaces (figure 2I).

### Rhizobium exhibits in vitro plant growth promotion mechanisms

The results of *in vitro* plant growth promotion analysis showed that strains PETP01 and TPV08 were not able to produce ACC deaminase or solubilize phosphate. Both strains grew on the CAS indicator medium but only the colonies of strain TPV08 were surrounded by a yellow-orange halo (3.5 mm radium around colonies) indicative of siderophore production. Both TPV08 and PETP01 strains were also able to grow in JMM medium supplemented with tryptophan producing similar amounts of indole acetic acid (75 m gl^−1^ and 63 m gl^−1^, respectively). Therefore both strains presented a direct mechanism for plant growth promotion (indole acetic acid production) and siderophore production by strain TPV08 additionally implies the ability of this strain to assist in iron acquisition (Table 1).

### Rhizobium inoculation promotes growth of pepper and tomato

The results of seed inoculation assays showed that TPV08 and PETP01 promote growth of both tomato and pepper. The dry weight of the inoculated seedlings (shoots and roots) was more than twice with respect to the uninoculated seedlings (Table 2).

**Table 2:** Results of the inoculation on tomato and pepper seedlings.

Treatment	Shoot dry weight (mg) (± S.E.)*	Root dry weight (mg) (± S.E.)*
**Tomato**		
Control	9.0 (±0.2)^a^	2.3 (±0.1)^a^
TPV08	23.1 (±0.3)^b^	6.0 (±0.1)^b^
PETP01	26.2 (±0.3)^b^	5.5 (±0.1)^b^
**Pepper**		
Control	16.4 (±0.4)^c^	3.5 (±0.1)^c^
TPV08	34.0 (±0.2)^d^	7.2 (±0.1)^d^
PETP01	33.2 (±0.5)^d^	6.5 (±0.1)^d^

Values followed by the same letter in each treatment are not significantly different from each other at *P* = 0.05 according to Fisher's Protected LSD (Least Significant Differences). S.E. = Standard Error.

* From each treatment 15 seedlings were included in this analysis.

The results of long term microcosm assays (Table 3) showed an increase in flowers and fruits number at harvest in tomato plants inoculated with PETP01 that was significant in the case of the flower number compared to the uninoculated plants. Non significant differences were found in the case of strain TPV08 in these two parameters. Although differences were not significant, the fresh fruit weight from tomato plants inoculated with both strains increased on about 10%. Significant differences in the percentage of N, P, K and Mg were found when TPV08 was inoculated and in P, K and Mg when PETP01 was the inoculated strain (Table 3).

**Table 3:** Results from microcosm experiment in tomato and pepper.

Treatment	Flowers/plant (± S.E.)*	Fruits/plant (± S.E.)*	Fresh weight/fruit (g) (± S.E.)¥	N (%) (± S.E.)*	P (%) (± S.E.)*	K (%) (± S.E.)*	Mg (%) (± S.E.)*	Ca (%) (± S.E.)*
**Pepper**								
Control	21 (±2.8)^a^	4 (±0.8)^a^	34.8 (±2.8)^a^	2.28 (±0.01)^a^	0.33 (±0.02)^a^	2.16 (±0.06)^a^	0.18 (±0.01)^a^	0.09 (±0.01)^a^
TPV08	28 (±1.8)^b^	10 (±1.2)^b^	44.7 (±2.4)^b^	2.33 (±0.08)^a^	0.36 (±0.01)^a^	2.15 (±0.03)^a^	0.18 (±0.01)^a^	0.10 (±0.01)^a^
PETP01	22 (±3.2)^ab^	6 (±0.7)^ab^	41.9 (±2.6)^b^	2.28 (±0.07)^a^	0.35 (±0.01)^a^	2.20 (±0.01)^a^	0.19 (±0.03)^a^	0.14 (±0.03)^a^
**Tomato**								
Control	55 (±8.6)^c^	21 (±3.4)^c^	5.47 (±0.4)^c^	1.41 (±0.03)^c^	0.36 (±0.01)^c^	2.09 (±0.03)^c^	0.14 (±0.02)^c^	0.07 (±0.04)^c^
TPV08	55 (± 11.1)^c^	19 (±4.2)^c^	6.04 (±0.9)^c^	1.92 (±0.08)^d^	0.43 (±0.43)^d^	2.48 (±0.01)^d^	0.17 (±0.01)^d^	0.06 (±0.03)^c^
PETP01	71 (±10.1)^d^	38 (±8.2)^cd^	6.04 (±0.6)^c^	1.43 (±0.01)^c^	0.41 (±0.01)^d^	2.50 (±0.01)^d^	0.16 (±0.01)^d^	0.11 (±0.04)^c^

Values followed by the same letter in each treatment are not significantly different from each other at *P* = 0.05 according to Fisher's Protected LSD (Least Significant Differences). S.E. = Standard Error.

* Flowers and fruits were counted in 25 plants per treatment.

¥ Results from 25 mature fruits per treatment at harvest (10 weeks).

The results on pepper showed a significant increase in the number of flowers and fruits at harvest when the plants were inoculated with strain TPV08 and a less significant increase in the case of strain PETP01 compared to the control. The fresh weight of fruits was significantly higher in inoculated than in uninoculated pepper plants. The increase in the fresh weight of pepper fruits ranged from 20 to 30% when the plants were inoculated with TPV08 and PETP01 strains, respectively, compared to the uninoculated control (Table 3).

## Discussion

Raw consumed foods must be free of harmful microorganisms. Therefore, in no circumstances, risk 2 pathogens such as *B. cepacia*, *P. aeruginosa* or *A. baumanii*, can be used in biofertilization schemes. Fortunately, many rhizospheric species are classified as risk 1 and thus they are non-pathogenic for humans as occurs with bacteria currently commercialised as biofertilizers. Rhizobia used for more than 100 years in legume biofertilization [22] are particularly safe for humans and since they presented direct and indirect mechanisms of plant growth promotion they are also excellent candidates to be used for non-legume biofertilization particularly of raw consumed vegetables [23], [28], [30]. However, up to date there was a significant lack of research about the growth promotion of rhizobia on vegetables with edible fruits such as pepper and tomato, the plants analysed in the present study.

Most rhizobial strains promoting non-legume plant growth described to date belong to biovars phaseoli and trifolii from *R. leguminosarum* [23], [26], [28], [30]. Therefore in this study we selected two strains phylogenetically related to this species on the basis of the *recA* and *atpD* gene analysis (figure 1) that belong to the biovars phaseoli (strain TPV08) and trifolii (strain PETP01) as revealed by the *nodC* gene (figure S1). These strains presented a direct mechanism of *in vitro* plant growth promotion, indole acetic acid production, and strain TPV08 was additionally able to produce siderophores as an indirect mechanism. Both mechanisms have been commonly reported in rhizobia (Table 1) and similar levels of indole acetic and siderophore production have been reported for strains *R. leguminosarum* P31 and R1 that were able to colonize maize and lettuce roots [23], [31]. Nevertheless to effectively promote plant growth bacteria should be able to colonize roots [4]. In the case of the legumes, rhizobial root colonization constitutes a crucial step in the infection process [32] and the attachment of bacteria is followed by the establishment of microcolonies by clonal propagation which is the initial step in biofilm formation [33]. In the case of non-legumes the colonization of lettuce roots by *Rhizobium* has been reported [31], but there are no studies in other raw consumed vegetables. Therefore this is the first report on the colonization of tomato and pepper roots by strains of genus *Rhizobium*.

This ability together with the production of several compounds involved in plant growth promotion make our strains good candidates to perform *in planta* experiments. These experiments should be carried out in different production steps in the case of tomato and pepper because they involved different producers, nurseries that commercialise seedlings and farmers that commercialise the fruits. The effect of seed bacterization was always positive since the seedlings of tomato and pepper were longer than those from uninoculated controls and these results agree with those of other reports where *Rhizobium* is able to stimulate the shoot growth of plants [23], [26], [28], [29]. In the case of the fruits, the inoculation effect was also positive in both tomato and pepper, although in pepper was more linked to the fruit production, with significant increases in the fresh weight of fruits, and in the case of tomato was more linked to their quality since significant increases were found in the percentage of N, P, K or Mg. These results are in agreement with those found in lettuce and sunflower in which the inoculation with *R. leguminosarum* strains led to an increase in N and P uptake [23], [29]. Therefore *Rhizobium* strains are excellent biofertilizers for tomato and pepper in different production steps leading to increased yield and quality.

Our results extend the number of plants susceptible to be biofertilized with rhizobia to vegetables appreciated for their fruits (Table 1). These results are crucial because it is essential to have plant growth promoting bacteria safe for humans to be used as biofertilizers especially for raw consumed vegetables. Infections caused by bacteria that may contaminate fresh vegetables could cause serious health problems and we should not forget that leaves, roots, fruits and even some seeds of non-legumes are raw consumed. Since nowadays producers aim not only to reach the maximal yielding of crops but also the maximum quality of final product, the safety of rhizobia, demonstrated after several decades of legume inoculation, ensures that they are optimal bacteria for biofertilization.

## Materials and Methods

### Phylogenetic classification of strains used in this study

The strains used in this study were previously isolated from nodules of *Trifolium pratense* [34] and *P. vulgaris* [35]. The phylogenetic location of these strains was analysed on the basis of the *atpD* and *recA* housekeeping genes amplified and sequenced as was previously described [36] and the biovar analysis was based on *nodC* symbiotic gene amplified and sequenced as was previously described [37]. These sequences were aligned with those of other species from genus *Rhizobium* using the Clustal W software [38]. The distances were calculated according to Kimura's two-parameter model [39]. Phylogenetic trees were inferred using the neighbour-joining method [40]. Bootstrap analysis was based on 1000 resamplings. The MEGA 4 package [41] was used for all analyses.

### GFP-labelling of strains

To express GFP from the broad-host range vector pBBR1MCS-2 [42], the coding sequence was transferred from pAcGFP-1 (Clontech, Palo Alto, CA, U.S.A.), using unique *EcoRI* and *SalI* restriction sites into similarly digested pBBR1MCS-2. The resulting plasmid pMRGFP was transferred from *E. coli* DH5α cells to TPV08 and PETP01 strains by triparental mating using the helper plasmid pRK2013 [43].

Plasmid pHC60 [44] was introduced into TPV08 and PETP01 strains by conjugation using *E. coli* S17.1 [45] as donor strain. For these matings, fresh cultures of donor and recipient strains were mixed on YMA plates and incubated overnight at 28°C. Transconjugants were selected on *Rhizobium* minimal medium (Rm) [46] plates supplemented with the corresponding antibiotics (kanamycin at 50 µg/ml and tetracyclin at 10 µg/ml, respectively). Transfer of pMRGFP and pHC60 to strains TPV08 and PETP01 yielded bacteria expressing the expected GFP as detected by fluorescence microscopy using a NIKON eclipse 8Oi fluorescence microscope.

These recombinant strains were routinely grown at 28°C in TY medium [47] supplemented with kanamycin (50 µg/ml) or tetracycline (10 µg/ml).

### Plant colonization assays

Seeds of tomato var. “cherry” and pepper var. “verde italiano” were surface-sterilized by immersion in 70% ethanol for 30 seconds followed by 5% sodium hypochlorite aqueous solution during 15 min. Seeds were washed six times with sterile water, and were germinated in water agar plates overlaid with Whatman number 1 sterile paper wetted with sterile water. The plates were placed in darkness in a growth chamber at 24°C until the seedling roots were 1–2 cm.

The GFP-tagged strains were grown for 48 h at 28°C in the previously mentioned medium and then cells were washed twice and resuspended in sterile water at a final concentration of 10^8^ cells/ml. The seedlings of tomato and pepper were inoculated with 1 ml of this suspension.

The seedlings were maintained in the dark and the roots were observed five days post inoculation with the rhizobial strains. Unbound bacteria were removed by gently washing the roots three times with sterile distilled water before microscopic observation. Uninoculated roots of tomato and pepper were included in the experiment as negative controls. Confocal spectral microscopy was carried out with a Leica SP5 confocal microscope equipped with a krypton–argon laser using a blue excitation filter (excitation maximum 488 nm; 530-nm long-pass filter). Projections were made from adjusted individual channels in the image stacks using Leica software as was previously described [48].

### Analysis of plant growth promotion mechanisms in vitro

In this study four mechanisms of *in vitro* plant growth promotion were analysed: solubilization of phosphate and siderophore, indole acetic acid and ACC deaminase production. The solubilization of insoluble phosphate was analysed on YED-P plates containing 2% CaHPO_4_ that were incubated for 15 days at 28°C [25]. Siderophore production was evaluated in M9-CAS-AGAR [49] modified with the addition of a cationic solvent, HDTMA, to stabilise the Fe-CAS complex providing the characteristic colour [50]. Indole acetic acid production was evaluated in JMM medium [51] supplemented with 0,17 gl^−1^ of tryptophan. After 7 days incubation the supernatants were recovered by centrifugation at 5000×g and filtered using 0.22 µm Millipore filters (Millipore Co., Amicon, USA). Then 1 ml of Salkowsky agent was added to 2 ml of supernatant and the red colour formed was measured by spectrophotometry at 550 nm using an ATI Unicam 8625 Spectometer (Mattson, USA) [52]. ACC deaminase production was tested in JMM medium [51] supplemented with 3 µM of ACC [53].

### Growth promotion assays in plants

Plant growth promotion was evaluated on tomato var. “cherry” and pepper var “verde italiano” in two steps, seedlings and fruit production. A total of 40 non-sterilised seeds per treatment were germinated in peat following the steps commonly used in the commercial process of seedling production and were irrigated from a bottom reservoir with water every 48 h and with commercial Hoagland's solution (Sigma Co., USA) every 8 days. Once placed on peat, each seed was inoculated with 1 ml of a suspension (2×10^8^ CFU/ml) of 5-days-old *Rhizobium* strains grown on YMA. Then they were covered with vermiculite and germinated in darkness in a growth chamber at 24°C for four days in the case of tomato and seven days in the case of pepper. Then they were maintained in a plant growth chamber with mixed incandescent and fluorescent lighting (400 microeinsteins m^−2^ s^−1^; 400 to 700 nm), programmed for a 16 h photoperiod, day-night cycle, with a constant temperature varying from 25–27°C, and 50–60% relative humidity. Uninoculated controls of both tomato and pepper were maintained in the same conditions. After four weeks 15 plants per treatment were dried and the dry weight per plant of shoots and roots was measured.

The remaining 25 plants per treatment were used for a microcosm experiment, which was performed in a soil collected in a zone that has clayey soils with neutral pH, and 1.5–1.8% of organic matter and 0.09–0.1 of N content in Salamanca (Spain). The seedling roots were flooded in bacterial suspensions containing 2×10^8^ CFU/ml during 4 h. Each plant was transplanted to a pot containing 2 Kg soil. Plants were irrigated from a bottom reservoir with water every 48 h and with commercial Hoagland's solution (Sigma Co., USA) every 8 days. The plants were maintained during 10 weeks in a greenhouse illuminated with natural light in summer (night temperature ranging from 15 to 20°C and day temperature ranging from 25 to 35°C) with humidity control. At the end of the experiment, flowers and fruits were counted, fruits were harvested and their fresh weight was measured. Flowers and fruits were counted in 25 plants per treatment and fresh weight was obtained in 25 mature fruits per treatment at harvest (10 weeks). The analysis of N, P, K, Ca and Mg were performed in the Ionomic Service from CEBAS-CSIC (Spain). Statistical analyses were carried out using StatView program for Macintosh computers. Data were analyzed by one-way analysis of variance, and mean values compared by Fisher's Protected LSD test (Least Significant Differences) (P≤0.05).

## Supporting Information

Figure S1 **Neighbour-joining phylogenetic tree based on** ***nodC*** **gene sequences (540 nt) showing the position of strains PETP01 and TPV08.** Bootstrap values calculated for 1000 replications are indicated. Bar, 5 nt substitution per 100 nt.
(TIF)
