# Supplementary figures and images for: Rhizobium Promotes Non-Legumes Growth and Quality in Several Production Steps: Towards a Biofertilization of Edible Raw Vegetables Healthy for Humans
Source: PLoS One. 2012 May 31;7(5):e38122. doi: 10.1371/journal.pone.0038122 (PMC3364997; doi:10.1371/journal.pone.0038122)

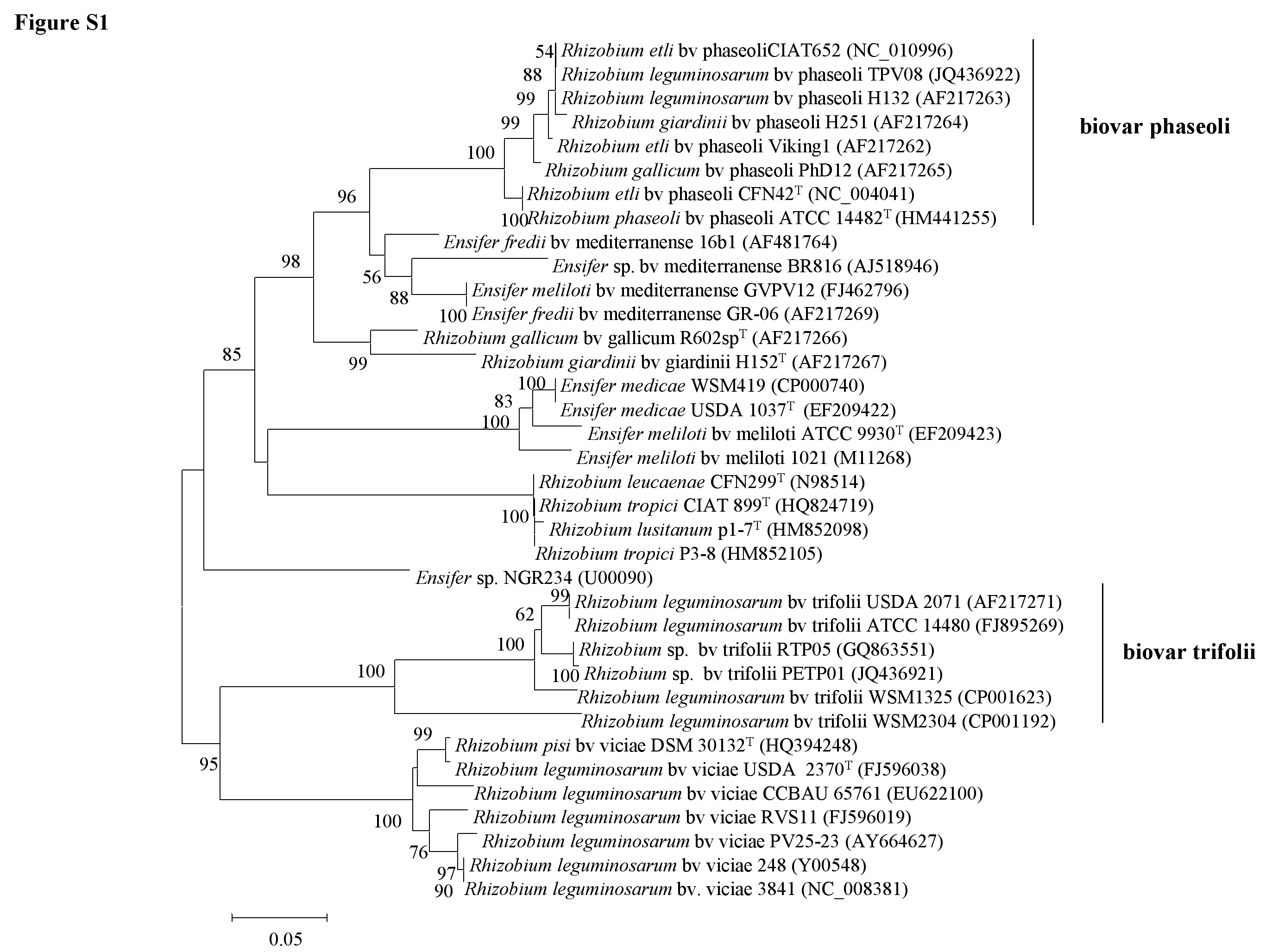

Supplement: Figure S1 — Neighbour-joining phylogenetic tree based on nodC gene sequences (540 nt) showing the position of strains PETP01 and TPV08. Bootstrap values calculated for 1000 replications are indicated. Bar, 5 nt substitution per 100 nt. (TIF) [file pone.0038122.s001.tif]
